# Psychrotrophic yeast *Yarrowia lipolytica* NCYC 789 mediates the synthesis of antimicrobial silver nanoparticles via cell-associated melanin

**DOI:** 10.1186/2191-0855-3-32

**Published:** 2013-06-07

**Authors:** Mugdha Apte, Devashree Sambre, Shital Gaikawad, Swanand Joshi, Ashok Bankar, Ameeta Ravi Kumar, Smita Zinjarde

**Affiliations:** 1 Institute of Bioinformatics and Biotechnology, University of Pune, Pune, 411007, India

**Keywords:** *Yarrowia lipolytica*, Silver nanoparticles, Melanin, Antibiofilm activity

## Abstract

A psychrotrophic marine strain of the ascomycetous yeast *Yarrowia lipolytica* (NCYC 789) synthesized silver nanoparticles (AgNPs) in a cell-associated manner. These nanostructures were characterized by UV-Visible spectroscopy and scanning electron microscope-energy dispersive spectrometer (SEM-EDS) analysis. The brown pigment (melanin) involved in metal-interactions was obtained from the cells. This extracted pigment also mediated the synthesis of silver nanoparticles that were characterized by a variety of analytical techniques. The melanin-derived nanoparticles displayed antibiofilm activity. This paper thus reports the synthesis of AgNPs by the biotechnologically important yeast *Y. lipolytica*; proposes a possible mechanism involved in the synthetic process and describes the use of the bio-inspired nanoparticles as antibiofilm agents.

## Introduction

It is a well established fact that nanoparticles of noble metals (gold and silver) exhibit strong antimicrobial properties (Zhou et al. 2012). An increase in the price of gold in the recent past has brought some restriction on the use of nanoparticles of this metal for antimicrobial applications. On the other hand, antimicrobial activity of silver has been documented since a very long time. In the past few decades, the significance of silver nanoparticles (AgNPs) as antimicrobial agents has increased to a great extent (Rai et al. 2009). Compared to metallic silver, AgNPs display higher surface to volume ratio and exhibit enhanced antimicrobial activity. AgNPs are extensively used in the field of medicine (Jain et al. 2009; Ravindra et al. 2010).

The conventional processes for nanoparticle synthesis involve top-down or bottom-up approaches usually employing physical or chemical protocols. However, such processes are often energy-demanding and cost-intensive (Zinjarde, 2012). Moreover, the chemicals used in such procedures are toxic and the size of the nanoparticles is difficult to control. In view of these problems, biological systems have emerged as effective alternatives for the clean and green synthesis of nanostructures (Sharma et al. 2009; Thakkar et al. 2010; Gade et al. 2010). *Yarrowia lipolytica* is a yeast that is routinely used in the synthesis of citric acid, mannitol and erythritol (Rywińska and Rymowicz 2010; Tomaszewska et al. 2012). The yeast is also associated with different cheese varieties (Lanciotti et al. 2005; Gardini et al., 2006). In some cases, this causes the brown discoloration of cheese (Carreira et al. 1998). The brown color is due to a pigment, melanin (Carreira et al. 2001). It is a well-known fact that fungal melanins have strong anti-oxidant properties that protect them from radiation, high temperature, free radical attack and metals (Fogarty and Tobin 1996 Apte et al. 2013).

During the present study, we put forth the following hypotheses (i) The yeast *Y. lipolytica* may produce silver nanoparticles (ii) melanin maybe involved in nanoparticle synthesis and (iii) the bio-inspired AgNPs may display antimicrobial activity against pathogenic bacteria. To test the aforementioned hypotheses, we chose a psychrotrophic strain of *Y. lipolytica* (NCYC 789) hitherto not reported to synthesize AgNPs. This paper describes the conditions for the synthesis of AgNPs (by cells and by melanin isolated from the cells) and their characterization by a variety of analytical techniques. In addition, applications of the melanin-mediated silver nanoparticles as antibacterial and antibiofilm agents against a representative pathogen are also demonstrated.

## Materials and methods

### Microorganism and culture maintenance

A psychrotrophic strain of *Y. lipolytica* deposited in National Collection of Yeast Cultures, U.K. as NCYC 789 was used in this study. Stock cultures of the yeast were maintained on MGYP slants containing malt extract, 3.0; glucose, 10.0; yeast extract, 3.0; peptone, 5.0; agar, 25.0 g/l of distilled water and sub-cultured at monthly intervals. The culture was grown at 20°C.

### Synthesis of silver nanoparticles by *Y. lipolytica* NCYC 789

Yeast cells were inoculated in YNB glucose (yeast nitrogen base, 7.0; glucose, 10.0 g/l distilled water) and incubated on a shaker (130 rpm, 48 h, 20°C). The culture broth was centrifuged (6000 × *g*, 10 min, 10°C) and the cell pellets were washed thrice to remove residual glucose. Fixed numbers of cells (10^10^cells/ml estimated by counting on a Neubauer chamber) were re-suspended in 20 ml of aqueous AgNO_3_ solutions to achieve final concentrations of 1.0, 2.0, 3.0 or 4.0 mM. The mixtures were incubated in dark on a shaker (130 rpm, 120 h, 20°C). Aliquots were withdrawn and the contents were centrifuged as described above. The re-suspended cell pellet and the cell-free medium were monitored for the presence of intracellular or extracellular silver nanoparticles, respectively, by measuring the UV-Visible spectra. All experiments were carried out in triplicates and representative data is presented here.

### Extraction of melanin from yeast cells

The melanin was extracted by following the standard procedure described earlier (Ito et al. 2007 Apte et al. 2013). Briefly, yeast cells were grown at 20°C for 72 h and the biomass was harvested by centrifugation (8000 × *g*, 15 min, 10°C). The washed cell pellets were dried in an oven to constant weight. Ethanol (10 ml) was added to 600 mg of dry cells and the test tubes were incubated at 60°C for 3 h. The contents were centrifuged, washed twice with distilled water and dried for 24 h at 95°C. The pellet was resuspended in 4 ml of 6 mol/l HNO_3_ and heated at 70°C for 3 h. This mixture was centrifuged and the supernatant was removed. The pellet was washed twice with distilled water. The melanin was extracted by boiling in 10 ml of 0.5 mol/l NaOH for 20 min. The resulting solution was passed through a 0.22 μ membrane filter to remove undigested debris and quantified by referring to a standard graph.

### Synthesis of AgNPs by melanin

AgNPs were synthesized by incubating extracted melanin with silver nitrate solutions. To check the effect of melanin content on nanoparticle synthesis, varying concentrations of the pigment (100, 150, 250, 500 μg) were incubated with 1 ml of 2.5 mM AgNO_3_ solutions. To enhance the reaction, the mixture was heated at 100°C for 10 min. Visual changes and UV–vis spectra were recorded. To check the effect of silver nitrate on nanoparticle synthesis, 2.5, 3.3, 5.0 and 10.0 mM of the silver salt were incubated with 500 μg/ml of melanin. All experiments were carried out in triplicates with two biological replicates and representative data is present here.

### Characterization of the AgNPs

The reaction mixtures were monitored over a period of time and visual observations were recorded. Cell control reactions lacked the silver salt and salt controls lacked the biomass. A Jasco V-530 spectrophotometer was used for UV-Visible spectroscopy measurements. AgNPs synthesized by the extracted melanin were also characterized similarly. Washed yeast cells or the melanin-mediated AgNPs were coated onto glass pieces and thoroughly dried. XRD measurements of thin films of yeast cells exposed to AgNO_3_ were carried out in the transmission mode on a D8 Advanced Brucker instrument with Cu Kα radiation using λ = 1.54˚A. SEM-EDS analysis was performed on air-dried, platinum-coated cell samples using an analytical SEM (JEOL JSM-6360A) equipped with EDS. All these samples were also analyzed in triplicates and representative micrographs are included here. In order to determine the functional groups involved in nanoparticle synthesis, FTIR spectroscopy was carried out as described earlier (Bankar et al. 2010a.) The melanin-derived AgNPs were also characterized in a similar manner. For transmission electron microscope (TEM) observations, samples were coated on carbon-coated copper grids (200 μm × 200 μm mesh size) and images were obtained on a TECNAI G2 20U-Twin (FEI, Netherlands) electron microscope.

### Antimicrobial and antibiofilm activities of nanoparticles

Antimicrobial activity of the melanin-mediated AgNPs against a representative pathogen *Salmonella paratyphi* MTCC 735 was checked by the disc diffusion method as described earlier (Bankar et al. 2010b). The extracted melanin was used as a control. The plates were incubated at 37°C for 24 h and zones of inhibition were checked. The effect of nanoparticles on biofilm formation by *S. paratyphi* in microtitre plates was also evaluated. To each well, 200 μl of nutrient broth, NB (containing yeast extract, 3.0; peptone, 5.0; sodium chloride, 5.0 g/l of distilled water), 5 μl of culture (10^6^ cells/ml) and 20 μl nanoparticles (derived from reaction mixtures containing 500 μg of melanin and 5.0 mM AgNO_3_) were added. In control wells, the nanoparticles were excluded. After incubation for 24 or 48 h, the supernatant was removed and plates were washed with phosphate buffer saline. The biofilm growth was stained with crystal violet, and quantification was done as reported earlier (Dusane et al. 2011). In the graph related to this data, mean values of triplicate experiments are plotted and error bars indicate standard deviation.

The antibiofilm activity of the AgNPs on a glass surfaces was also evaluated. In sterile Petri dishes, 20 ml NB, 5 μl of culture (10^6^cells/ml) and 200 μl of different concentrations of AgNPs (derived from reaction mixtures containing 500 μg of melanin and 2.5, 5.0 or 10.0 mM AgNO_3_) were added. Appropriate control experiments were also performed. The Petri plates were incubated for 48 h at 37°C after which, the slides were washed with distilled water, stained with acridine orange and observed under a fluorescent microscope (Axio Scope-A1 equipped with photographic attachment ProgRes ® Capture Pro 2.7 and software AxioVison Rel. 4.8).

## Results

### Visual observations and UV-Visible spectroscopic studies

*Y. lipolytica* cells synthesized AgNPs when incubated with silver nitrate. Control cells without AgNO_3_ did not show a characteristic brown color. Since cell pellets were brown and the cell-free supernatants did not display any color, it was concluded that the AgNPs were cell-associated. The UV-visible spectra of reaction mixtures after 120 h showed characteristic peaks at around 410 nm (data not shown). As the content of the silver salt was increased, there was a gradual increase in the intensity of the color and the peaks.

### Characterization of nanoparticles by XRD and SEM-EDS

**Figure 1:**
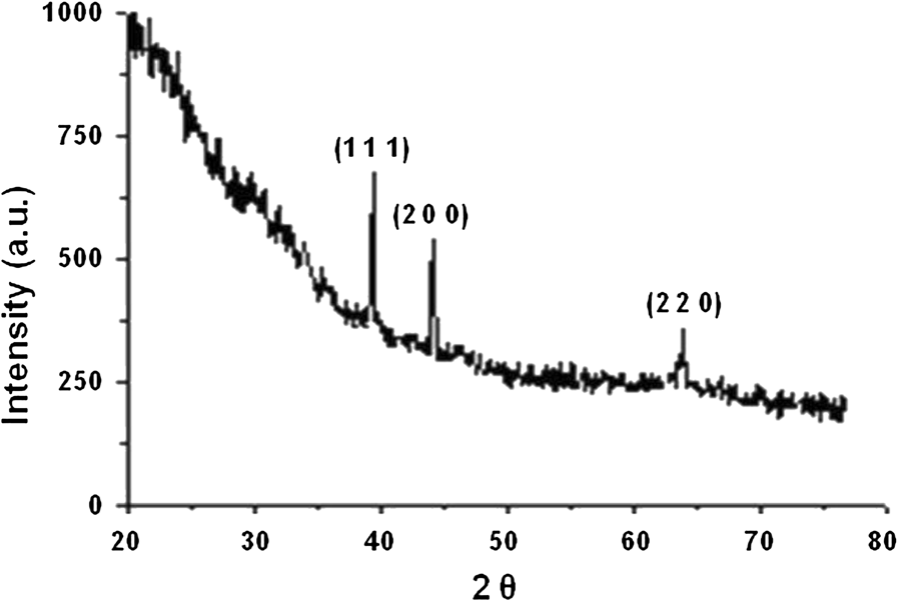
**Representative XRD pattern of silver nanoparticles synthesized by *Y. lipolytica *NCYC 789.**

A representative XRD pattern of thin films of *Y. lipolytica* NCYC 789 that had accumulated AgNPs is shown in Figure 1. The cell controls and the salt controls did not show the characteristic response. Bragg reflections at 2θ = 38.09°, 44.5°and 64.21° respectively, could be indexed to (111), (200) and (220) facets of the face-centered cubic metal silver structures (JCPDS No. 04–0784).

**Figure 2:**
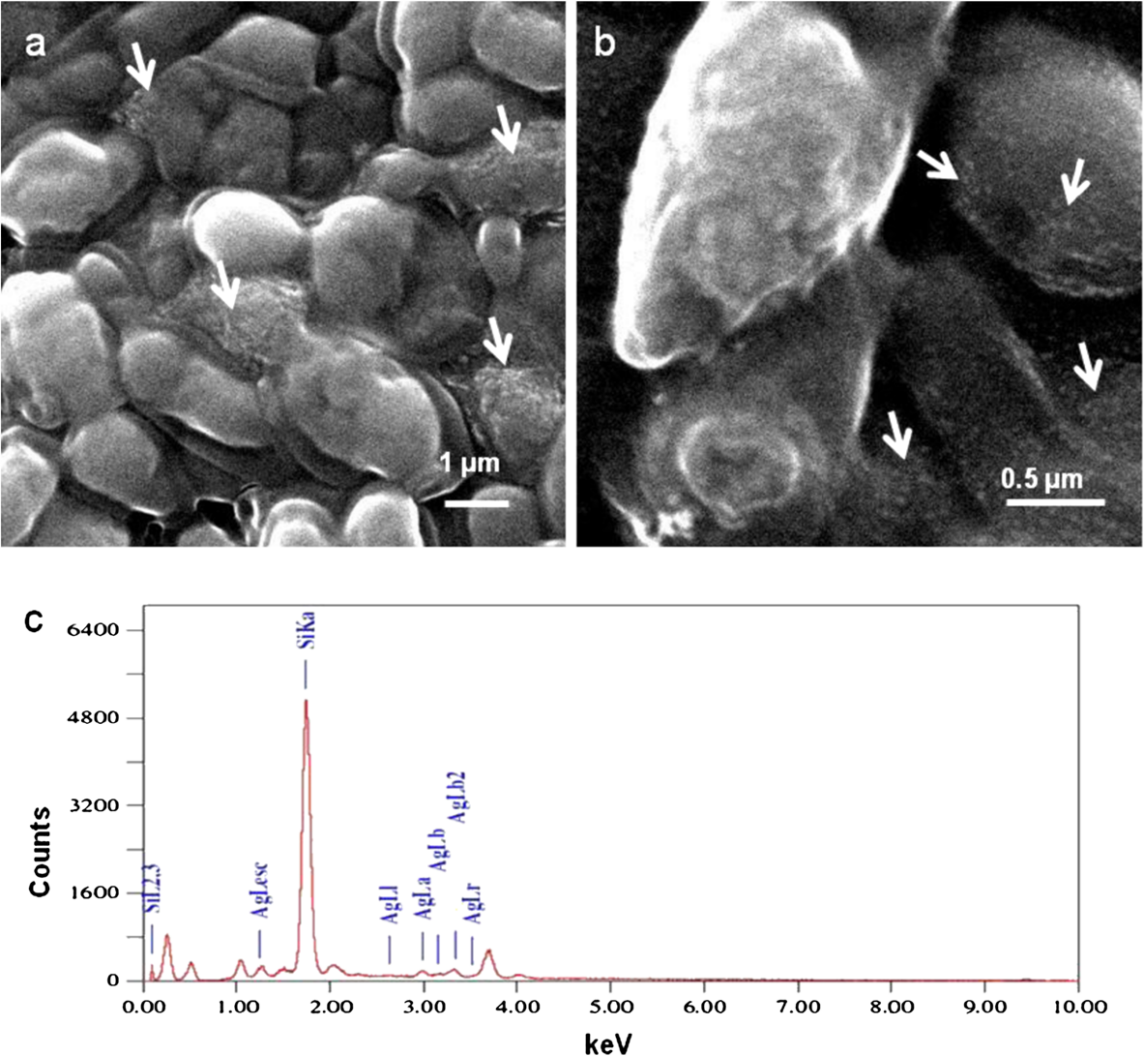
**Representative SEM images of *Y. lipolytica *NCYC 789 (after incubating 10**^**10 **^**cells/ml with 3.0 mM silver nitrate for 120 h), magnified (a) 10,000 X (b) 30,000X.** White arrows indicate the location of silver nanoparticles on the cells (**c**) Representative field EDS profile of the nanostructures.

Figure 2a and b show representative SEM images of *Y. lipolytica* NCYC 789 that had accumulated silver. Control cells in the present investigation also did not show the presence of nanoparticles. Nanostructures were observed on the cell surfaces where silver ions were reduced to Ag° (Figure 2a and b, white arrows). Figure 2c depicts a representative spot energy dispersive spectrum indicating the presence of silver in the cell-associated nanostructures. Signals for Si (from the glass slide used in sample preparation) were also observed. From the above SEM images and the EDS spectra, the synthesis of AgNPs by the yeast culture was confirmed.

### FTIR analysis

**Figure 3:**
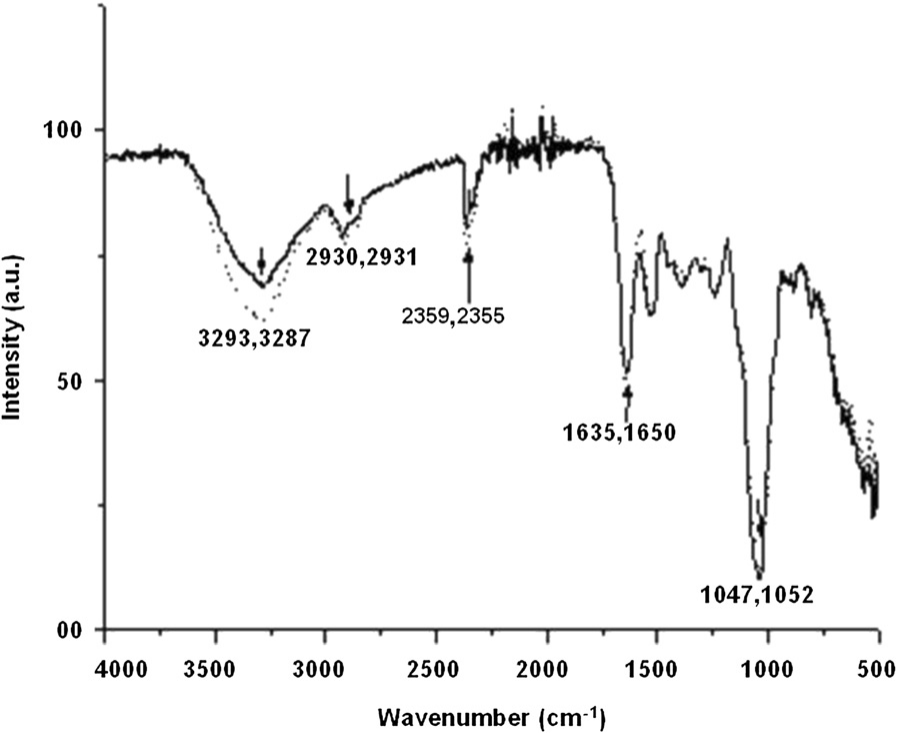
**Representative FTIR spectra of *Y. lipolytica *NCYC 789 cells before (**^**___**^**) and after AgNP synthesis (**^**…….**^**).**

The FTIR profiles of control (solid line) and silver-loaded test cells (dotted line) are shown in Figure 3. The spectra of the unexposed cells showed the presence of several peaks suggesting the complex nature of the yeast cell surface. There was a change in the intensity of the bands after interaction with silver (Figure 3, black arrows). The stretching vibrations of NH group shifted from 3293 cm^-1^ to 3287 cm^-1^. The peaks related to carboxylic or phenolic groups shifted from 2930 cm^-1^ to 2931 cm^-1^. The absorption peak of the amide-I group shifted from 1635 cm^-1^ to 1650 cm^-1^ and that of OH shifted from 2359 cm^-1^ to 2355 cm^-1^. The peak related to the C-N group shifted from 1047 cm^-1^ to 1052 cm^-1^. The data thus suggested the involvement of phenolic, hydroxyl and amino groups in the nanoparticle synthetic process.

On the basis of these results and the fact that cell-associated silver nanoparticles have limited applications, a set of experiments was designed to isolate and extract the cell-associated biopolymer, melanin and to employ this in AgNP synthesis. Melanin isolated from cells was used to synthesize AgNPs in a form which was free of the cells. The effect of varying concentrations of melanin on AgNP synthesis was studied. A yellow color was observed with 100 and 150 μg of melanin while characteristic brown colors were observed with 250 and 500 μg of melanin when 2.5 mM of AgNO_3_ was used. The UV-Visible spectra also displayed characteristic peaks at around 410 nm for these concentrations. The effect of silver nitrate concentration on nanoparticle synthesis was also studied. As the concentration of silver salt was increased, the intensity of the brown color gradually increased.

### SEM-EDS and TEM analysis of melanin mediated silver nanoparticles

**Figure 4:**
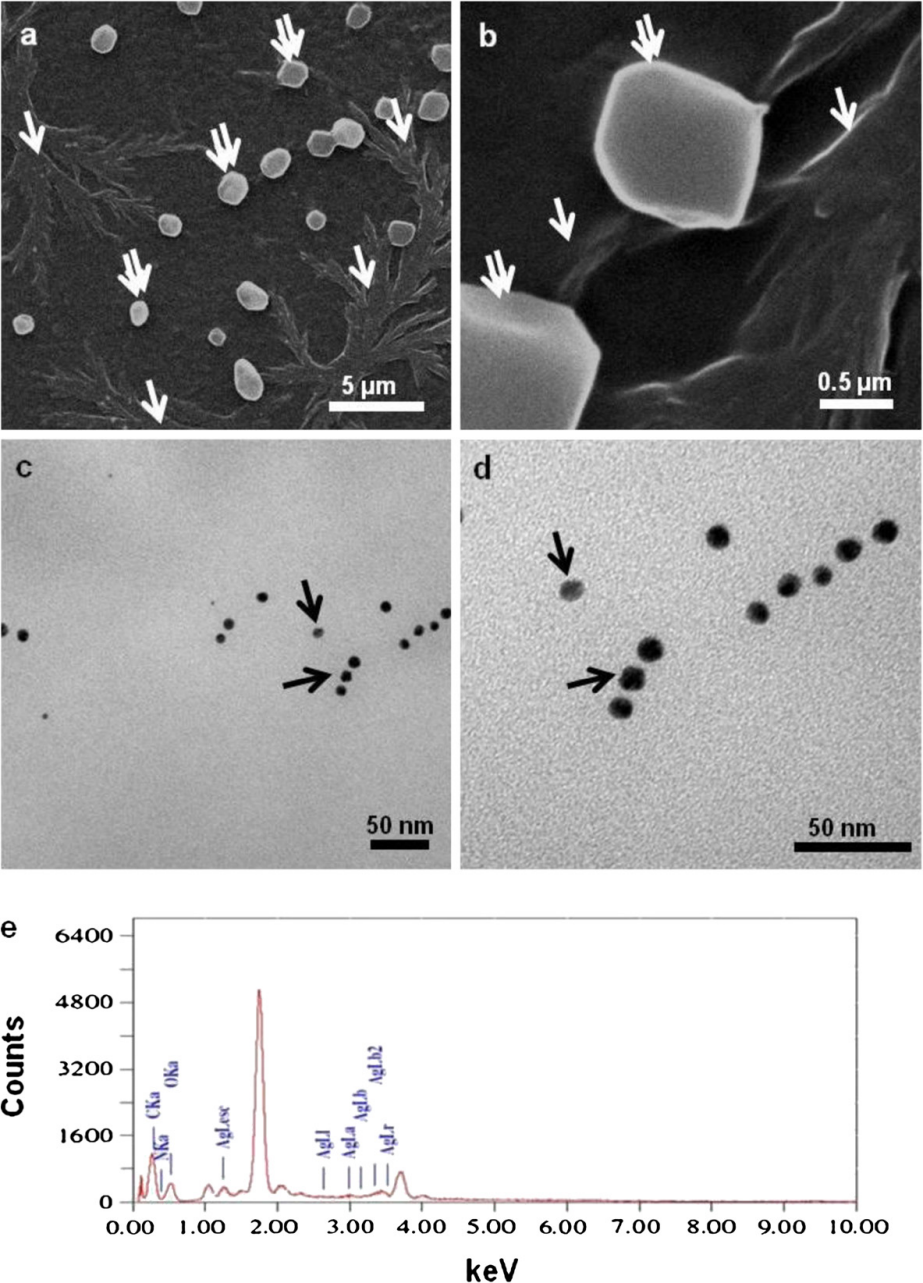
**Representative electron microscopic images of silver nanoparticles synthesized by melanin after incubating 500 μg of melanin with 2.5 mM silver nitrate for 10 min at 100°C.** SEM images magnified **(a)** 4,000 X **(b)** 30,000 X **(c)** and **(d)** TEM images at different magnifications **(e)** representative field SEM-EDS profile of the nanostructures.

Representative SEM images of the melanin-mediated nanostructures magnified 4,000 and 30,000 times, respectively are depicted in Figure 4a and b. The nanoparticles aggregated into dendrites (single arrows) and large structures (double arrows) as an outcome of sample preparation step (air-drying on glass slides). TEM images on the other hand showed the monodisperse nature of the nanoparticles with an average size of about 15 nm (Figure 4c and d black arrows). The SEM-EDS attachment confirmed that the structures were made of silver (Figure 4e).

**Figure 5:**
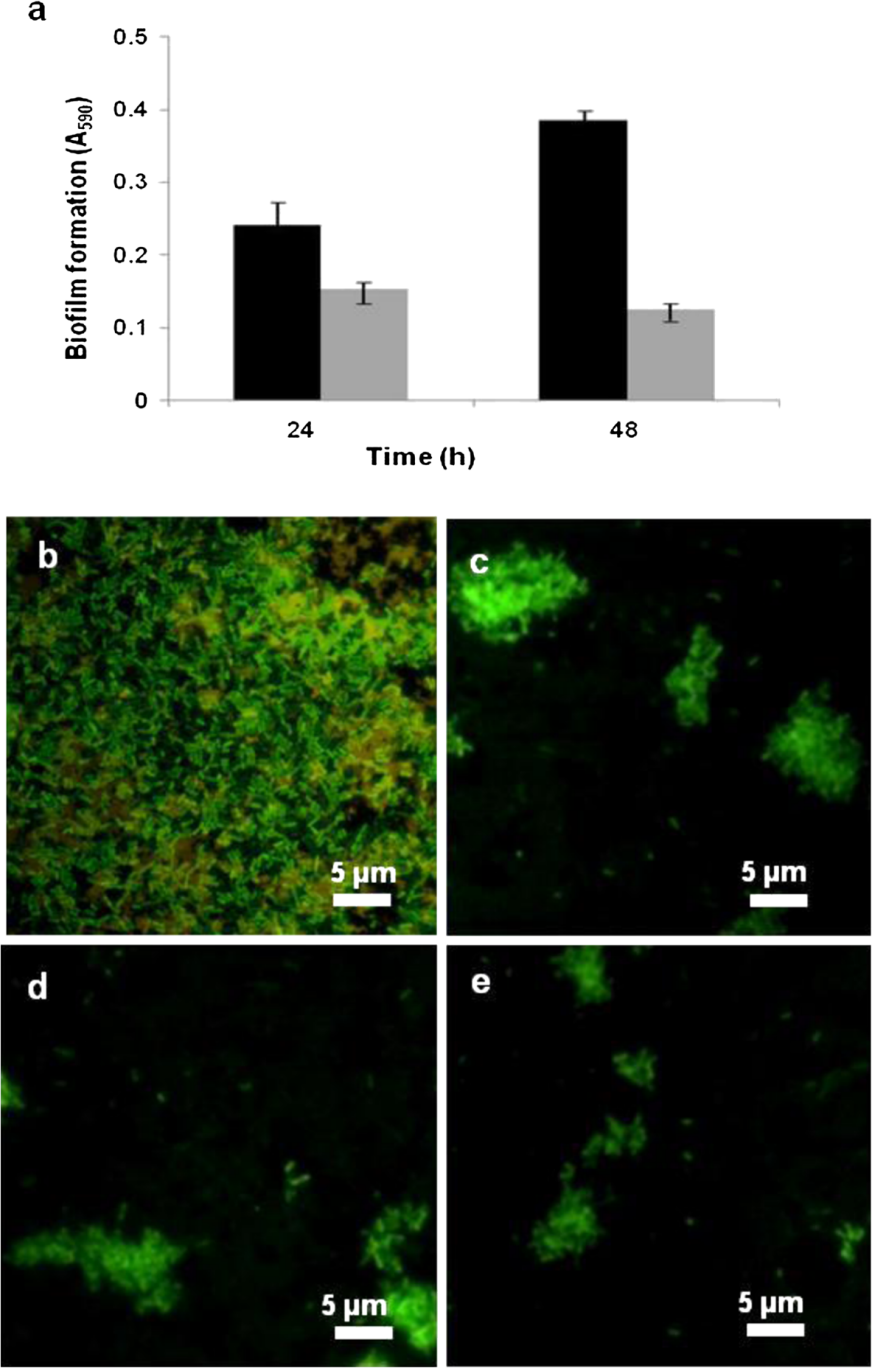
**Representative results of antibiofilm activities of the melanin mediated silver nanoparticles (a) Inhibition of *Salmonella paratyphi *biofilms by AgNPs in microtitre plates (control: black bars; test: gray bars).** Fluorescent microscopy images of *S. paratyphi* biofilms **(b)** control, **(c)**, **(d)**, **(e)** with 2.5, 5.0, 10.0 mM AgNPs, respecively.

Cell-associated silver nanoparticles are often difficult to separate prior to their subsequent use. The melanin-mediated AgNPs on the other hand were in the cell-free form. They were tested for their antibacterial and antibiofilm activity. Clearance zones were observed on plates seeded with *S. paratyphi* MTCC 735 cultures that had been exposed to melanin-mediated AgNPs (data not presented). These nanoparticles were thus effective antibacterial agents. The melanin-derived AgNPs were also evaluated for their antibiofilm potential against a representative pathogen, *S. paratyphi* in microtitre plates and on glass surfaces. Figure 5a summarizes the data on the inhibition of *S. paratyphi* biofilms on polystyrene surfaces (microtitre plates) by melanin-mediated AgNPs. Black bars in the figure represent control biofilms (not treated with AgNPs) and grey bars depict biofilm inhibition after treatment with AgNPs. From the figure, it is evident that the AgNPs inhibited the proliferation of biofilms by 37% and 67%, after 24 h and 48 h, respectively. The effect of AgNPs with respect to biofilm inhibition on glass surfaces was more pronounced. Figure 5b shows a representative image of the control biofilm of *S. paratyphi* developed in the absence of the AgNPs. The glass surface was extensively covered with the biofilm growth. However, biofilm formation was inhibited when AgNPs were included (Figure 5c,d and e).

## Discussion

Different strains of *Y. lipolytica* play an important role in biotechnology (Fickers et al. 2005; Beopoulos et al. 2010). A tropical marine isolate of *Y. lipolytica* has been reported to mediate the synthesis of gold nanoparticles (Agnihotri et al. 2009; Apte et al. 2013). Some strains of this yeast are also psychrotrophic in nature (Ross and Morris 1965; Margesin et al. 2003) Psychrophiles and psychrotrophs are an important group of microorganisms that have several important applications (Feller and Gerday 2003; Bankar et al. 2009; Sa´nchez et al. 2009). Recently, there has been a report on the synthesis of silver nanoparticles by psychrophilic bacteria (Shivaji et al. 2011). To the best of our knowledge there are no reports on the synthesis of nanoparticles by cold-adapted yeasts and thus this investigation was taken up. The psychrotrophic strain of *Y. lipolytica* NCYC 789 synthesized silver nanoparticles in a cell-associated manner. Some fungi such as *Phaenerochaete chrysosporium* and *Neurospora crassa* are also reported to synthesize nanostructures in this manner (Vigneshwaran et al. 2006; Castro-Longoria et al. 2011). The XRD profiles confirmed the crystalline nature of the silver nanoparticles associated with the cells. The peaks could be indexed to the (111), (200) and (220) facets of crystalline silver (Figure 1). *Lactobacillus fermentum* mediated silver nanoparticles are also known to exhibit such patterns (Sintubin et al. 2009). SEM observations in the current investigation match with those of other microbial systems that synthesize nanoparticles in a cell-associated manner. For example there is a report on *Penicillium* sp. reducing silver and gold ions to nanostructures on the cell surface similar to the present results (Du et al. 2010).

The significance of different functional groups in nanoparticle synthesis has been reported earlier. In the metal-reducing bacterium, *Shewanella oneidensis* MR-1, carbonyl, hydroxyl, amide and carboxyl were involved in the nanoparticle synthetic process (Suresh et al. 2010). In the current investigation, the nature of the cell surface was characterized and functional groups involved in AgNP synthesis were determined by FTIR analysis. The involvement of phenolic, hydroxyl and amino groups in nanoparticle synthesis was implicated.

As stated earlier, strains of *Y. lipolytica* are known to produce melanin (Carreira et al. 1998; Carreira et al. 2001). In addition, this pigment plays an important role in the sequestration of heavy metals and in the synthesis of gold nanoparticles (Bankar et al. 2009; Apte et al. 2013). We hypothesized that cell-associated melanin maybe mediating the reduction of silver ions to elemental silver nanoparticles on account of the following properties displayed by melanins (i) They are natural biopolymers of phenolic compounds (Jacobson, 2000) (ii) they exhibit strong anti-oxidant properties (Commoner et al. 1954; Doering et al. 1999) and (iii) the quinonic residues present in melanin can interchange between the hydroxyquinone and quinone forms (Horak and Gillette 1971). It is a well-established fact that hydroxyquinone-quinone transformations mediate nanoparticle synthesis (Duran et al. 2011). In the current study, the FTIR spectra of the control and silver-laden cells revealed that phenolic, hydroxyl and amide groups are involved in the process of AgNPs synthesis (Figure 3). The SEM images also indicated a cell-surface location of the silver nanostructures (Figure 2a and b, white arrows). Since melanin is known to be associated with cell wall of fungi (Jacobson, 2000), we thought that this pigment may be contributing towards AgNP synthesis in this yeast as well. The isolated melanin also mediated the synthesis of silver nanoparticles. The observations on the effect of salt concentration on nanoparticle synthesis by the yeast cells as well as by the isolated melanin are consistent with earlier results on the use of other biological material for AgNP synthesis (Bankar et al. 2010b). Although the size was determined to be 15 nm on the basis of the TEM images, in SEM images the AgNPs aggregated into dendrites and large structures as an outcome of the sample preparation procedure (Figure 4a and b). Silver nanoparticles synthesized by physical and chemical methods have an inherent property to assemble into larger structures (He et al. 2003; Tang et al. 2009).

**Figure 6:**
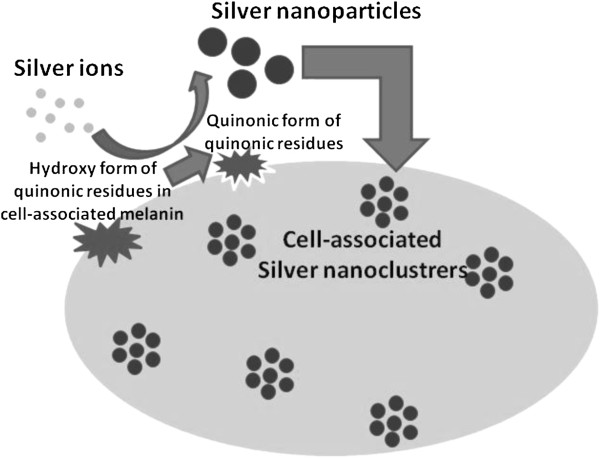
Proposed mechanism by which melanin mediates the synthesis of AgNPs.

Although there are a number of reports on the microbial synthesis of nanoparticles, a few of them describe the involvement of specific biomolecules in the process. In this regard, nitrate reductase along with a protein from *Aspergillus niger* and nitrate reductase and rhamnolipids from *Pseudomonas aeruginosa* are reported to be involved in nanoparticle synthesis (Gade et al. 2008; Kumar and Mamidyala 2011). There is also a report on the role of certain proteins in the cell-free supernatants of the mangrove associated fungi *Aspergillus tubingensis* and *Bionectria ochroleuca* in mediating the synthesis of silver nanoparticles (Rodrigues et al. 2013). In lactic acid bacteria, at high pH the glucose ring residues associated with the cell surface open to the aldehyde from and provide the reducing equivalents. In the presence of silver ions the aldehyde is oxidized to the corresponding carboxylic acid, and the metal ions are reduced to their elemental form (Sintubin et al. 2009). To the best of our knowledge, the significance of cell-associated melanin in the synthesis of AgNPs has not been reported earlier. Figure 6 depicts the possible manner in which melanin derived from *Y. lipolytica* mediated the synthesis of AgNPs. The quinonic residues of the melanin associated with the cell surface could alternate between the hydroxyl and the quinonic forms. During this transition, silver ions were reduced to AgNPs. There are earlier reports on polyphenolic compounds such as tannic acid mediating metal reductive reactions in a similar manner (Sivaraman et al. 2009).

Effectiveness of silver nanoparticles as antibacterial agents has been analyzed by a variety of techniques (Kim et al. 2011). Since several pathogenic bacteria exhibit the biofilm mode of growth (Hoiby et al. 2010), the role of the silver nanoparticles as antibiofilm agents was investigated. The antibiofilm activity displayed by the melanin induced AgNPs are consistent with the observations on other types of nanoparticles being effective against pathogens such as *P. aeruginosa* and *Staphylococcus epidermidis* (Kalishwaralal et al. 2010). The melanin-inspired AgNPs were thus effective in inhibiting the biofilm growth of a representative pathogen. The AgNPs obtained in the cell-free form could be in the future functionalized by a variety of techniques to enhance their antimicrobial activity (Veerapandian and Yun 2011).

In conclusion, we state that the biotechnologically significant yeast could effectively synthesize AgNPs. We have described the role of a natural biopigment (melanin) in the synthetic process. Cell-associated nanoparticles often pose problems during subsequent applications. This method of using melanin permitted the synthesis of nanoparticles in the free form. The melanin derived AgNPs were effective at disrupting biofilms of a representative pathogen *S. paratyphi* on polystyrene as well as glass surfaces. These nanoparticles are being differentially functionalized to enhance their antimicrobial activity.

## Competing interests

The authors declare that they have no competing interests.
